# Physical Therapy Interventions for Gait and Balance in Charcot-Marie-Tooth Disease: A Scoping Review

**DOI:** 10.3390/life15071036

**Published:** 2025-06-29

**Authors:** Roberto Tedeschi, Danilo Donati, Federica Giorgi

**Affiliations:** 1 Independent Researcher, 40100 Bologna, Italy; 2 Physical Therapy and Rehabilitation Unit, Policlinico di Modena, 41125 Modena, Italy; danilo.donati@unimore.it; 3 Clinical and Experimental Medicine PhD Program, University of Modena and Reggio Emilia, 41121 Modena, Italy; 4 Pediatric Physical Medicine and Rehabilitation Unit, IRCCS Institute of Neurological Sciences, 40139 Bologna, Italy; federica.giorgi15@gmail.com

**Keywords:** Charcot-Marie-Tooth disease, rehabilitation, walking capacity, balance training, neuromuscular disorders

## Abstract

**Background**: This scoping review aims to map and summarise physical therapy interventions specifically targeting gait and balance in individuals with Charcot-Marie-Tooth disease (CMT), highlighting commonly applied strategies, methodological limitations, and clinical implications. Charcot-Marie-Tooth disease (CMT) is a hereditary neuropathy characterised by progressive motor and sensory impairment, often resulting in reduced mobility, muscle weakness, balance deficits, and fatigue. Although pharmacological options remain limited, rehabilitation is increasingly recognised as a key component of disease management. However, the scope, type, and effectiveness of rehabilitative interventions in CMT remain poorly mapped. **Methods**: This scoping review was conducted in accordance with the Joanna Briggs Institute (JBI) methodology and the PRISMA-ScR guidelines. Five databases (PubMed, Cochrane, PEDro, Scopus, and Web of Science) were systematically searched up to March 2024. Studies were eligible if they involved participants with CMT undergoing rehabilitation interventions aimed at improving functional outcomes. Data extraction focused on study characteristics, methods, outcome measures, and results. **Results**: Eleven studies met inclusion criteria, comprising case reports, cohort studies, and two randomised controlled trials. Interventions included aerobic training, strength and balance exercises, videogame-based home programmes, and multidisciplinary rehabilitation. Most studies reported improvements in walking capacity (e.g., 6MWT, 10MWT), postural balance (e.g., BBS), and lower limb strength (e.g., MRC, dynamometry). Some also showed positive changes in fatigue and quality of life, though data were limited. Methodological heterogeneity and small sample sizes limited comparability and generalisability. **Conclusions**: Rehabilitation appears to yield meaningful improvements in key functional domains in people with CMT. Tailored, multimodal interventions show promise, though long-term benefits remain underexplored. Future research should adopt standardised protocols and outcome measures to better define best practices and optimise patient care.

## 1. Introduction

Charcot-Marie-Tooth disease (CMT) is one of the most prevalent inherited peripheral neuropathies, affecting approximately 1 in 2500 individuals worldwide [1,2]. It is characterised by progressive degeneration of peripheral nerves, predominantly affecting motor and sensory fibres, and is associated with a wide range of clinical manifestations, including distal muscle weakness, sensory deficits, foot deformities, and impaired gait and balance [3,4]. Although the disease course varies significantly among patients, CMT often leads to long-term disability and a substantial reduction in quality of life, particularly due to its impact on mobility and postural stability [5,6].

Despite extensive advances in the genetic characterisation and pathophysiology of CMT—currently over 130 causative genes have been identified—there are still no curative pharmacological treatments available [7]. Management is thus predominantly supportive and rehabilitation-based, with a strong emphasis on physiotherapy aimed at preserving motor function, compensating for neuromuscular deficits, and preventing secondary complications [8,9]. However, the heterogeneity of disease phenotypes and progression patterns complicates the standardisation of therapeutic interventions.

While numerous studies have explored the role of physiotherapy in managing CMT, the scientific literature remains fragmented, with considerable variability in intervention types, duration, outcome measures, and study design [10,11,12,13]. Some studies suggest improvements in gait endurance, static and dynamic balance, and functional capacity following rehabilitation protocols, including strength training, proprioceptive exercises, or treadmill-based interventions [14,15,16,17]. Yet, consensus is lacking regarding the optimal physiotherapeutic strategies, frequency and intensity of treatment, and sustainability of clinical gains over time. Furthermore, few reviews have systematically mapped and critically appraised the available evidence, focusing specifically on interventions targeting gait and balance, which are arguably among the most disabling aspects of the disease [18,19,20].

In this context, a comprehensive synthesis of existing evidence is urgently needed to identify promising rehabilitative approaches and to guide future clinical and research efforts. The present study addresses this gap by conducting a scoping review to map the current landscape of physiotherapy-based interventions in CMT, with a particular focus on outcomes related to ambulation and postural control. The aim of this scoping review is to map the existing literature on physical therapy interventions specifically targeting gait and balance in individuals with CMT in order to identify commonly applied approaches, assess the quality and consistency of protocols, and support the development of evidence-informed clinical guidelines. In the present review, the term “rehabilitation” encompasses both conventional and technology-assisted physiotherapy approaches. Conventional interventions include aerobic training, muscle strengthening, and balance exercises, while technology-based strategies involve videogame-based home programmes, treadmill devices, and robotic supports [9,21]. Pharmacological interventions, including disease-modifying or symptomatic drugs, were not considered in this review, as the focus was strictly on physical therapy strategies aimed at functional improvement. This clarification aims to define the scope of the present work and to support the rationale: mapping the existing evidence on physical therapy strategies (irrespective of technological support) to guide the development of tailored, non-pharmacological interventions for individuals with CMT.

## 2. Methods

This scoping review was developed following the methodological approach recommended by the Joanna Briggs Institute (JBI) [22] for conducting scoping reviews. To ensure methodological transparency and robustness, the process adhered to the Preferred Reporting Items for Systematic Reviews and Meta-Analyses extension for Scoping Reviews (PRISMA-ScR) guidelines [23].

### 2.1. Review Question

We formulated the following research question: “*What physiotherapy-based interventions are most effective in improving gait and balance in individuals diagnosed with Charcot-Marie-Tooth disease?*”

### 2.2. Eligibility Criteria

Studies were eligible for inclusion if they met the following Population, Concept, and Context (PCC) criteria.

**Population (P):** Individuals of any age diagnosed with Charcot-Marie-Tooth disease (regardless of subtype or disease stage), presenting documented impairments in balance and/or gait. Studies were eligible regardless of gender or geographical origin.

**Concept (C):** Interventions involving physiotherapy or physical therapy, either as a standalone approach or in combination with other therapeutic modalities, aimed specifically at improving gait and/or postural balance. Both conventional physiotherapy protocols and innovative or technology-assisted rehabilitation strategies were included.

**Context (C):** Any healthcare or rehabilitation setting, including hospital-based, outpatient, community, or home-based environments.

Only full-text articles published in peer-reviewed scientific journals were included in this review; abstracts, conference proceedings, and grey literature were excluded to ensure higher methodological robustness. No time restrictions were applied during the initial search to allow for comprehensive data mapping. However, the final included studies ranged from 2006 to March 2024. Furthermore, the IEEExplore database was not included in our search strategy, as our primary focus was on clinical rehabilitation outcomes rather than engineering or technical system development, which are more prevalent in IEEExplore.

The review protocol was not pre-registered. Based on the clarification of terminology, this review focuses exclusively on physical therapy interventions targeting ambulation and balance. Multidisciplinary rehabilitation strategies not primarily delivered by physiotherapists were excluded. The terms “physical therapy” and “physiotherapy” were not used interchangeably in the final screening stage to avoid conceptual overlap.

### 2.3. Exclusion Criteria

The following studies were excluded:

- Studies involving patients with comorbid neurological disorders not related to Charcot-Marie-Tooth disease.
- Studies that did not assess both gait and balance outcomes.
- Studies investigating orthotic or surgical interventions without an associated physiotherapeutic programme.
- Protocols, ongoing trials, abstracts without full text, and studies published in languages other than English (if not translatable).
- Reviews or opinion papers that did not include original clinical data or outcome measures.

### 2.4. Search Strategy

A comprehensive search strategy was implemented across five major biomedical databases, without restrictions on publication date or geographic setting. Search terms were adapted to each database’s indexing system and included both controlled vocabulary (e.g., MeSH) and free-text terms.

- MEDLINE (PubMed):
  (“charcot marie tooth disease” [MeSH Terms] OR “charcot marie tooth” [All Fields] OR “hereditary motor and sensory neuropathy” [All Fields] OR HMSN) AND (“rehabilitation” [MeSH Terms] OR “physiotherapy” [All Fields] OR “physical therapy modalities” [MeSH Terms] OR “exercise therapy”) AND (“gait” [MeSH Terms] OR “walking” [MeSH Terms] OR “balance” [MeSH Terms] OR “postural balance”)
- Cochrane Central:
  (charcot marie tooth OR hereditary motor and sensory neuropathy) AND (rehabilitation OR physiotherapy OR physical therapy) AND (gait OR walking OR balance)
- Scopus:
  TITLE-ABS-KEY (“charcot marie tooth” OR “hereditary motor and sensory neuropathy”) AND TITLE-ABS-KEY (“rehabilitation” OR “physiotherapy” OR “physical therapy”) AND TITLE-ABS-KEY (“gait” OR “balance” OR “walking”)
- PEDro:
  The search within the PEDro (Physiotherapy Evidence Database) platform was conducted using the advanced search function. The term “charcot marie tooth” was entered in the “Title & Abstract” field, and the subdiscipline was restricted to “Neurology” to enhance the specificity of the results. To ensure a comprehensive retrieval of relevant studies, an alternative search was also performed using the term “hereditary motor and sensory neuropathy” in the same field, again under the neurology subdiscipline. The search was not limited by publication type, language, or date, and all study designs available in the PEDro database were considered eligible. Due to the constraints of the platform, Boolean operators such as AND/OR could not be applied across multiple fields simultaneously. Therefore, broader terms were used, and the relevance of each record was verified manually through title and abstract screening. All retrieved records were exported and catalogued using Zotero for subsequent de-duplication and eligibility assessment.
- Web of Science:
  TS = (“charcot marie tooth” OR “hereditary motor and sensory neuropathy”) AND TS = (“rehabilitation” OR “physiotherapy” OR “physical therapy”) AND TS = (“gait” OR “walking” OR “balance”)

### 2.5. Study Selection

The identification and selection of studies were carried out through a structured and methodical process, consistent with established standards for scoping reviews. All retrieved records were imported into Zotero, where duplicates were eliminated. Screening was conducted in two sequential phases: an initial review of titles and abstracts, followed by a full-text assessment of potentially relevant studies. Both phases were independently undertaken by two reviewers, with disagreements resolved through consultation with a third reviewer. The entire procedure was guided by the PRISMA 2020 recommendations to uphold methodological transparency and quality. As this is a scoping review aimed at mapping the existing literature rather than assessing the quality or efficacy of interventions, protocol registration was not mandatory. Therefore, the review was not prospectively registered.

### 2.6. Data Extraction and Data Synthesis

Key information—such as study design, participant characteristics, intervention specifics, measured outcomes, and principal results—was systematically extracted using a predefined data collection form to ensure consistency across included studies. Extracted outcomes were grouped into categories to facilitate comparative evaluation. A qualitative synthesis was employed to detect recurring themes and highlight existing gaps in the current evidence base. Where applicable, quantitative findings were summarised to underscore relevant trends. This structured method allowed for a thorough and coherent integration of the available data in alignment with the study’s objectives.

## 3. Results

**Figure 1:**
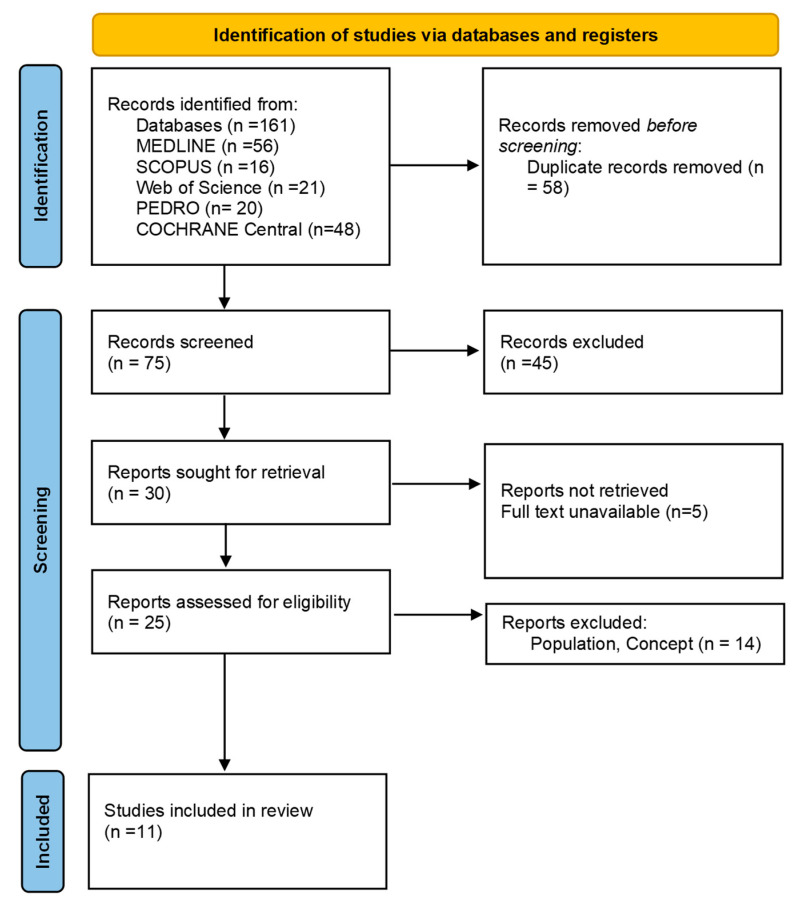
Preferred reporting items for systematic reviews and meta-analyses 2020 (PRISMA) flow diagram.

As illustrated in the PRISMA 2020 flow diagram (Figure 1), the initial database search yielded a total of 161 records. Following the screening and eligibility assessment, 150 studies were excluded based on the predetermined criteria, and 11 studies met the inclusion criteria and were retained for analysis (see Table 1 and Table 2).

### 3.1. Walking Performance

Walking performance was one of the most consistently assessed outcomes, primarily evaluated through the Six-Minute Walk Test (6MWT) and Ten-Metre Walk Test (10MWT). In the largest study included, Mori et al. reported that participants undergoing both the TreSPE and SPE protocols improved their 6MWT performance, with mean values rising from 320.3 ± 85.6 m at baseline (T0) to 351.9 ± 90.1 m at three months (T1), although no significant differences were found between groups. Ferraro et al. (2024) [9,27] observed significant improvements in 6MWT and 10MWT immediately following a three-week intensive rehabilitation programme, but these improvements regressed at the 12-month follow-up, returning to near-baseline levels. Pazzaglia et al. [13] demonstrated statistically significant increases in both 6MWT and Dynamic Gait Index (DGI) one month after focal mechanical vibration therapy. Bottoni et al. [16] found improvements in gait velocity and walking distance, with concurrent increases in plantar and dorsiflexor strength. Dudziec et al. [15] reported that participants in the intervention group improved their walking speed and Walk-12 scores compared to the control group. In paediatric cases, Burns et al. [24] and Pagliano et al. recorded gains in walking endurance, but improvements were modest and not statistically validated. Overall, walking performance appears to benefit from both aerobic and multimodal rehabilitation interventions, though long-term retention of gains is limited without sustained therapy.

### 3.2. Balance

Balance outcomes were assessed using multiple tools, most commonly the Berg Balance Scale (BBS), and showed widespread improvement across studies. Knak et al. [11] reported statistically significant BBS gains following a treadmill-based aerobic programme, despite minimal improvement in walking distance. Matjacić and Zupan [10] demonstrated marked improvements in BBS and Timed Up and Go (TUG) scores in both control and experimental groups, although gains were significantly greater in the group using the Balance Trainer device. In the study by Pazzaglia et al. [13], both BBS and DGI scores improved significantly at the one-month follow-up after focal mechanical vibrations. Kobesova et al. [12] used computerised dynamic posturography to detect significant gains in mCTSIB, Limits of Stability (LOS), and Forward Lunge (FL), which were also reflected in patients’ subjective perceptions. Ferraro et al. [9] (2024) showed significant short-term gains in BBS, though these were not sustained over time. Dudziec et al. [15] found strong post-treatment improvements in BBS, BESTest, and static posturography, and these were associated with reduced fear of falling. In a paediatric context, Pagliano et al. observed functional improvements in balance through BOT-2 scores after five weeks of interactive gaming-based training. Overall, balance was consistently responsive to both intensive and home-based interventions across all age groups.

### 3.3. Muscle Strength

Muscle strength, assessed via the Medical Research Council (MRC) scale and handheld dynamometry, improved across most interventions. Ferraro et al. (2024) [9,25] reported statistically significant improvements in MRC scores at the end of treatment, particularly in lower limb muscle groups; however, these gains diminished by the 12-month follow-up. In a post-surgical cohort, Ferraro et al. (2024) [9] documented significant recovery in proximal muscle groups after surgery and rehabilitation. Burns et al. [24] observed increased strength in dorsiflexors and plantarflexors following a 12-week home-based strengthening protocol, although no improvements were found in the evertor group. Bottoni et al. [16] recorded asymmetrical strength gains, with improvement in plantar and dorsiflexion on the left side and only partial gains on the right. Mori et al. [26] reported an increase in plantarflexor strength using dynamometry, though no between-group differences were specified. These findings suggest that strength can be enhanced across a wide range of intervention strategies, though targeted and symmetrical outcomes are more difficult to achieve in unilateral or progressive presentations.

### 3.4. Fatigue

Fatigue was assessed in three studies using the Fatigue Severity Scale (FSS) and the Checklist Individual Strength–Revised (CIS-20R). Knak et al. noted a reduction in FSS scores, though the changes did not reach statistical significance. Bottoni et al. [16] described a subjective reduction in fatigue perception, supported by lower CIS-20R scores, in a patient undergoing an adapted physical activity programme. Ferraro et al. (2024) [9,25] found a substantial decrease in perceived fatigue after an intensive rehabilitation cycle, although these improvements were not maintained at long-term follow-up. These results indicate that fatigue is a responsive but transient outcome and may require ongoing treatment strategies to maintain benefits.

### 3.5. Pain and Cramping

Pain and cramping, assessed by the Visual Analogue Scale (VAS) and Verbal Rating Scale (VRS), showed variable but generally positive responses. Ferraro et al. (2024) [9] observed significant reductions in pain following a protocol involving foot surgery and intensive rehabilitation. Ferraro et al. (2017) [25,28] also found that pain and muscle cramping diminished post-intervention, but these gains were not fully preserved after one year. Kobesova et al. reported a complete resolution of low back pain and substantial reduction in plantar foot pain, underscoring the value of multimodal approaches. While not all studies reported pain data, the available evidence supports the utility of structured physical therapy programmes for managing chronic musculoskeletal symptoms in patients with CMT.

### 3.6. Quality of Life and Patient-Reported Outcomes

Improvements in quality of life and patient-perceived function were commonly reported using the SF-36, Walk-12 scale, and HADS. In the study by Mori et al. [26], both protocols led to improvements in the physical component of the SF-36 and in walking-related quality of life (Walk-12), with TreSPE participants maintaining these improvements at six-month follow-up. Bottoni et al. [16] also noted increased well-being and reduced fatigue perception, supported by SF-36 domain scores. Dudziec et al. found improved scores in Walk-12, reduced anxiety and depression (HADS), and lower fear of falling (FES-I) in the intervention group. Pazzaglia et al. [13] reported modest improvements in SF-36 and self-reported mobility outcomes. These findings suggest that quality of life benefits may parallel objective gains, particularly when interventions are personalised and sustained over time.

## 4. Discussion

This scoping review aimed to systematically identify and describe the current evidence on rehabilitation strategies for individuals diagnosed with Charcot-Marie-Tooth disease (CMT), focusing on key functional domains such as walking capacity, postural balance, muscle strength, fatigue, and overall quality of life (QoL). Eleven studies published between 2009 and 2024 were included, comprising case reports, case series, cohort studies, and two randomised controlled trials (RCTs). The interventions were heterogeneous in design and delivery, including aerobic treadmill training, progressive resistance exercises, functional electrical stimulation, videogame-based home programmes, and intensive multidisciplinary rehabilitation protocols. Despite the methodological diversity, a general trend towards improvement was observed in most of the evaluated outcomes.

Walking performance emerged as the most frequently assessed domain, with both the Six-Minute Walk Test (6MWT) and the Ten-Metre Walk Test (10MWT) being the most commonly used instruments. Improvements in walking endurance and speed were reported in 9 of the 11 studies. For instance, in the study by Bottoni et al. [16], the 6MWT distance increased from 513 m to 580 m over 8 months of adapted physical activity. Similarly, Ferraro et al. [9] documented an improvement in mean 10MWT time from 9.2 ± 2.1 s to 7.8 ± 1.9 s at the end of a 3-week intensive programme (*p* < 0.05). However, longitudinal follow-up data, such as those in Mori et al. [26], suggested that some improvements in gait parameters may diminish within 6 months post-intervention, highlighting the need for continuity in care and possibly maintenance programmes to prevent regression.

Postural balance was the second most commonly reported domain and showed a strong positive response to targeted interventions. The Berg Balance Scale (BBS), in particular, showed significant improvements across multiple studies. For example, in the study by Pazzaglia et al., mean BBS scores increased from 42 to 49 (*p* = 0.01) after three days of focal vibration therapy. Likewise, Matjacić and Zupan [10] demonstrated greater improvements in BBS and Timed Up and Go (TUG) scores in participants receiving physiotherapist-supervised balance training using a robotic platform compared to those receiving conventional therapy, with between-group differences reaching statistical significance (*p* < 0.05). Balance improvements were also corroborated by objective stabilometric data in Kobesova et al., reinforcing the potential of integrative sensory-motor rehabilitation even in more advanced CMT subtypes such as CMTX.

Muscle strength gains were reported in 7 studies, though not always consistently across all muscle groups. The use of Medical Research Council (MRC) grading, handheld dynamometry, or electromyographic (EMG) parameters enabled quantitative assessment of strength changes. For instance, Burns et al. [24] reported an increase in ankle dorsiflexor strength from 2.5/5 to 4/5 on the MRC scale following a 12-week progressive home-based strengthening protocol. In Ferraro et al. [25], post-surgical rehabilitation led to a mean increase of 0.5 points in MRC scores across lower limb proximal muscle groups, while distal musculature showed more modest gains. These findings suggest that while muscle strength can improve with targeted rehabilitation, the degree of recovery is influenced by disease subtype, chronicity, and baseline severity.

Fatigue, although less commonly investigated, was examined using validated tools such as the Fatigue Severity Scale (FSS) and CIS-20R. Bottoni et al. [16,29] observed a reduction in perceived fatigue scores by 1.8 points on the FSS after an 8-month intervention, while Ferraro et al. [9] (2024) reported significant within-group changes in VRS fatigue scores (*p* = 0.02), although the sustainability of these improvements at 12-month follow-up was questionable. Given that fatigue is one of the most disabling symptoms reported by individuals with CMT, the limited number of high-quality studies addressing this outcome represents a critical gap in the literature.

Quality of life outcomes, typically measured via the SF-36 or the Walk-12 scale, showed moderate improvements, particularly in physical functioning and vitality domains. For instance, Mori et al. [26] recorded a mean increase of 8 points in the SF-36 physical component summary score in the TreSPE group (*p* < 0.05). Likewise, in Dudziec et al. [15], patient-reported improvement in Walk-12 scores suggested a subjective perception of enhanced walking ability and confidence, even in the absence of large objective gains in gait metrics. Importantly, psychological outcomes such as fear of falling and anxiety (measured via HADS) also improved in some trials, indicating a multidimensional impact of rehabilitation.

A key observation of this review is the heterogeneity in rehabilitation approaches and the lack of standardised intervention protocols or outcome sets. While this diversity reflects the personalised nature of care in rare neuropathies, it also poses challenges for synthesis and comparison. Moreover, variations in study design (e.g., single-case reports vs. cohort trials), participant characteristics (age, disease subtype), and follow-up durations further complicate the interpretation of efficacy. Few studies adopted control groups or blinded assessors, raising concerns about internal validity. Similarly, adverse events were poorly reported, and adherence to treatment protocols was inconsistently monitored, limiting our ability to fully evaluate the feasibility and safety of the interventions.

Despite these methodological issues, the aggregated evidence points towards the potential utility of comprehensive, sustained, and multidisciplinary rehabilitation for improving function and QoL in individuals with CMT. Future research should aim to overcome current gaps through methodologically robust designs, longitudinal monitoring, and greater emphasis on patient-reported outcomes.

## 5. Limitations

Several limitations must be acknowledged in interpreting the findings of this scoping review. First, the overall number of studies remains small, and many are of limited methodological quality. A substantial proportion of the included literature comprises case reports or case series, which inherently carry a higher risk of bias and limited generalisability. Only two studies adopted randomised controlled designs, and long-term follow-up data were scarce.

Second, the diversity in intervention types, durations, and intensities complicates direct comparison across studies. For instance, some protocols focused exclusively on strength training, while others employed complex, multimodal regimens including hydrotherapy, Tai-chi, and occupational therapy. Additionally, the inclusion of paediatric and adult populations further adds to the heterogeneity, as baseline functional capacities and rehabilitation potential differ significantly between these groups.

Third, outcome measures were inconsistent. While tools such as the BBS and 6MWT were frequently used, others such as EMG analysis, gait labs, or high-fidelity dynamometry were reported only in isolated cases. Patient-reported outcomes, despite their clinical importance, were inconsistently applied and seldom used as primary endpoints. This lack of standardisation limits the reproducibility and external validity of the findings.

Moreover, reporting quality was often insufficient. Sample sizes were frequently underpowered, and several studies failed to report effect sizes, statistical significance, or confidence intervals. Adherence rates and adverse events were only sporadically documented, further constraining the evaluation of feasibility and safety.

One notable limitation of this review is the initial conceptual ambiguity between “physical therapy and “physical therapy”. While the two are often used interchangeably in the literature, they refer to distinct domains: the former encompassing multidisciplinary efforts, the latter referring specifically to interventions delivered by physiotherapists. This distinction was addressed and clarified during the data extraction and interpretation phases, but may have introduced variability in the initial screening process.

Finally, publication bias cannot be ruled out. Studies with positive results are more likely to be published, particularly in small populations with rare diseases such as CMT. This could lead to an overestimation of the true effect of rehabilitation interventions.

A limitation of this review is the potential conceptual overlap between multidisciplinary rehabilitation and physiotherapy; although we restricted inclusion to physical-therapy-led protocols, some primary studies used the broader term ‘rehabilitation’, which may have introduced heterogeneity.

## 6. Clinical Practice Implications

Despite these limitations, the findings of this review offer valuable insights for clinicians involved in the rehabilitation of individuals with CMT. First, they suggest that both structured institutional programmes and home-based interventions can lead to clinically meaningful improvements in mobility, balance, and strength. Clinicians should therefore consider integrating individualised exercise regimens into routine care, adapted to the patient’s functional level, disease severity, and access to resources.

Second, the review highlights the value of using standardised outcome measures in clinical practice. Tools such as the BBS, 6MWT, and patient-reported outcomes such as the SF-36 and Walk-12 scale can guide treatment goals, monitor progress, and facilitate communication among multidisciplinary teams. Where possible, integrating more objective measures such as handheld dynamometry or instrumented gait analysis may enhance the precision of assessment and tracking.

Third, the results support the concept of multimodal and interdisciplinary rehabilitation. Combining aerobic, strength, proprioceptive, and functional training appears more effective than single-modality approaches. Clinicians should thus advocate for comprehensive treatment plans that incorporate elements of physical therapy, occupational therapy, and even technological supports such as video games or wearable feedback systems, particularly in younger populations.

Finally, ongoing monitoring and follow-up appear crucial to maintaining gains. Several studies showed functional regression after cessation of treatment, underscoring the chronic and progressive nature of CMT. Rehabilitation should be envisioned not as a discrete intervention, but as a long-term process embedded within a continuum of care. This has implications for service planning, patient education, and resource allocation.

## 7. Conclusions

This scoping review demonstrates that targeted physical therapy programmes can improve gait and balance in CMT, although protocols and outcome measures remain heterogeneous. Despite heterogeneity in protocols and limited high-quality trials, most studies reported improvements in walking capacity, balance, muscle strength, and quality of life. These findings underscore the potential of structured, individualised, and multidisciplinary interventions to address key functional impairments in CMT. Future research should prioritise rigorous methodologies, standardised outcome measures, and long-term follow-up to strengthen clinical recommendations and optimise patient care.

## Figures and Tables

**Table 1:** Summary of included studies on rehabilitation interventions in patients with Charcot-Marie-Tooth Disease.

Authors, Year	Enrolled Patients	Experimental Protocol	Evaluation Metrics	Quantitative Results
Knak et al., 2017 [11]	5 patients (CMT1A = 4, CMTX = 1)	10-week control phase followed by 10-week treadmill aerobic training (30 min, 3x/week)	6MWT, BBS, stabilometric tests, FSS, SF-36	Improved BBS; walking non-significant; 2 cases of temporary pain
Bottoni et al., 2024 [16]	1 male, 16 y.o. (CMT1A)	Adapted motor activity (1 h twice/week, 8 months)	SF-36, CIS-20R, 6MWT, 10MWT, SPPB, BBS, YBT, MVC, TOE, EMG	Improved fatigue, balance, and walking; mixed muscle strength
Kobesova et al., 2012 [12]	1 male, 55 y.o. (CMTX)	3-week intensive rehab (2.5 h/day)	mCTSIB, LOS, FL (dynamic posturography)	Improved balance and walking stability; reduced plantar and back pain
Matjacić & Zupan, 2006 [10]	16 ambulant patients (CMT1)	2-week rehab: Balance Trainer vs. conventional physio	BBS, TUG, 10MWT	Significant improvements in both groups; greater with Balance Trainer
Ferraro et al., 2024 (1) [9]	37 patients (CMT1, CMT2, mixed)	3-week intensive rehab (2–4 h/day, 5x/week)	MRC, VRS, BBS, Walk12, 10MWT	Significant gains post-treatment; partial regression at 12-month follow-up
Pazzaglia et al., 2016 [13]	14 patients (CMT1A)	3-day focused mechanical vibration on lower limb muscles	BBS, DGI, 6MWT, MRC, stabilometry, SF-36	Improved BBS and DGI; mild changes in 6MWT, MRC, SF-36
Burns et al., 2009 [24]	1 female, 15 y.o. (AR-CMT2)	12-week dorsiflexor strengthening home programme (3x/week)	Dynamometry, BOT2, jump test, 6MWT, gait analysis	Strength gains in dorsiflexors; unchanged balance/endurance
Dudziec et al., 2024 [15]	14 patients (CMT1A, fall history)	12-week home-based exercise + fall education	BBS, BESTest, posturography, 10MWT, FGA, dynamometry, Walk12, SF-36, FES-I, IPAQ, HADS	Improved balance and walking in the intervention group; better subjective outcomes
Pagliano et al., 2018 [21]	1 male, 9 y.o. (CMT1A)	5-week home programe: ankle strengthening + Kinect balance games (3x/week)	BOT2, jump test, 6MWT, dynamometry, Walk-12	Improved balance and endurance; mixed strength outcomes
Ferraro et al., 2017 [25]	5 patients (CMT1A)	Foot surgery, 3 weeks in a cast, then 3-week of intensive rehab (2x/day, 5x/week)	BBS, WHS, 10MWT, Gait Analysis, Walk-12, VAS, MRC, OHS, CMTNS	Improved proximal strength, balance, and pain; limited gait gains
Mori et al., 2022 [26]	53 ambulant patients (CMT1A)	3-month TreSPE vs. SPE (treadmill, proprioception, etc.)	6MWT, 10MWT, BBS, SPPB, dynamometry, CMTNS, Walk-12, SF-36	Improved gait and plantarflexor strength; better balance in the TreSPE group

Legend: 6MWT: Six-Minute Walk Test; 10MWT: Ten-Metre Walk Test; AR-CMT2: Autosomal Recessive Charcot-Marie-Tooth Type 2; BBS: Berg Balance Scale; BESTest: Balance Evaluation Systems Test; BOT-2: Bruininks–Oseretsky Test of Motor Proficiency, Second Edition; CIS-20R: Checklist Individual Strength–Revised; CMT1A/CMT2/CMTX: Charcot-Marie-Tooth subtypes 1A, 2, and X-linked; CMTNS: Charcot-Marie-Tooth Neuropathy Score; DGI: Dynamic Gait Index; EMG: Electromyography; FGA: Functional Gait Assessment; FSS: Fatigue Severity Scale; FL: Forward Lunge; Gait Analysis: Instrumented Gait Assessment; HADS: Hospital Anxiety and Depression Scale; IPAQ: International Physical Activity Questionnaire; LOS: Limits of Stability; mCTSIB: Modified Clinical Test of Sensory Interaction on Balance; MRC: Medical Research Council scale for muscle strength; MVC: Maximal Voluntary Contraction; OHS: Oxford Hip Score; SF-36: Short Form Health Survey 36; SPPB: Short Physical Performance Battery; TOE: Time of Electromechanical Delay; TreSPE/SPE: Treadmill-Stretching-Proprioception-Exercise/Stretching-Proprioception-Exercise; TUG: Timed Up and Go test; VAS: Visual Analogue Scale; VRS: Verbal Rating Scale; Walk-12: 12-item Walking Scale; WHS: Walking Handicap Scale; YBT: Y-Balance Test.

**Table 2:** Summary table of study types and outcomes.

Author	Study Type	Walking	Balance	Strength	Fatigue	QoL
Knak et al. [11]	Case series	↑	↑	→	→	→
Bottoni et al. [16]	Case report	↑	↑	↑	↑	↑
Kobesova et al. [12]	Case report	↑	↑	→	→	↑
Matjacić and Zupan [10]	RCT	↑	↑	↑	→	→
Ferraro et al. (1) [9]	Cohort study	↑	↑	↑	↑	↑
Pazzaglia et al. [13]	Case series	↑	↑	→	→	↑
Burns et al. [24]	Case report	→	→	↑	→	→
Dudziec et al. [15]	RCT	↑	↑	→	→	↑
Pagliano et al. [21]	Case report	↑	↑	→	→	→
Ferraro et al. (2) [25]	Case series	↑	↑	↑	→	→
Mori et al. [26]	Cohort study	↑	↑	↑	→	↑

Legend: ↑ = Improvement; → = No significant change/Not reported.

## References

[1] Nagappa M., Sharma S., Taly A.B. (2025). Charcot-Marie-Tooth Disease. StatPearls.

[2] Van den Bergh P.Y.K., van Doorn P.A., Hadden R.D.M., Avau B., Vankrunkelsven P., Allen J.A., Attarian S., Blomkwist-Markens P.H., Cornblath D.R., Eftimov F. (2021). European Academy of Neurology/Peripheral Nerve Society Guideline on Diagnosis and Treatment of Chronic Inflammatory Demyelinating Polyradiculoneuropathy: Report of a Joint Task Force-Second Revision. Eur. J. Neurol..

[3] Pipis M., Rossor A.M., Laura M., Reilly M.M. (2019). Next-Generation Sequencing in Charcot-Marie-Tooth Disease: Opportunities and Challenges. Nat. Rev. Neurol..

[4] Padua L., Aprile I., Cavallaro T., Commodari I., La Torre G., Pareyson D., Quattrone A., Rizzuto N., Vita G., Tonali P. (2006). Variables Influencing Quality of Life and Disability in Charcot Marie Tooth (CMT) Patients: Italian Multicentre Study. Neurol. Sci..

[5] Vinci P., Serrao M., Millul A., Deidda A., De Santis F., Capici S., Martini D., Pierelli F., Santilli V. (2005). Quality of Life in Patients with Charcot-Marie-Tooth Disease. Neurology.

[6] Johnson N.E., Heatwole C.R., Dilek N., Sowden J., Kirk C.A., Shereff D., Shy M.E., Herrmann D.N., Inherited Neuropathies Consortium (2014). Quality-of-Life in Charcot-Marie-Tooth Disease: The Patient’s Perspective. Neuromuscul. Disord..

[7] Fridman V., Reilly M.M. (2015). Inherited Neuropathies. Semin. Neurol..

[8] Corrado B., Ciardi G., Bargigli C. (2016). Rehabilitation Management of the Charcot-Marie-Tooth Syndrome: A Systematic Review of the Literature. Medicine.

[9] Ferraro F., Calafiore D., Curci C., Fortunato F., Carantini I., Genovese F., Lucchini G., Merlo A., Ammendolia A., de Sire A. (2024). Effects of Intensive Rehabilitation on Functioning in Patients with Mild and Moderate Charcot-Marie-Tooth Disease: A Real-Practice Retrospective Study. Neurol. Sci..

[10] Matjacić Z., Zupan A. (2006). Effects of Dynamic Balance Training during Standing and Stepping in Patients with Hereditary Sensory Motor Neuropathy. Disabil. Rehabil..

[11] Knak K.L., Andersen L.K., Vissing J. (2017). Aerobic Anti-gravity Exercise in Patients with Charcot–Marie–Tooth Disease Types 1A and X: A Pilot Study. Brain Behav..

[12] Kobesova A., Kolar P., Mlckova J., Svehlik M., Morris C.E., Frank C., Lepsikova M., Kozak J. (2012). Effect of Functional Stabilization Training on Balance and Motor Patterns in a Patient with Charcot-Marie-Tooth Disease. Neuro Endocrinol. Lett..

[13] Pazzaglia C., Camerota F., Germanotta M., Di Sipio E., Celletti C., Padua L. (2016). Efficacy of Focal Mechanic Vibration Treatment on Balance in Charcot-Marie-Tooth 1A Disease: A Pilot Study. J. Neurol..

[14] Tedeschi R. (2024). Podological Analysis in Children with Neuromotor Disabilities. Reabil. Moksl. Slauga Kineziter. Ergoter..

[15] Dudziec M.M., Lee L.E., Massey C., Tropman D., Skorupinska M., Laurá M., Reilly M.M., Ramdharry G.M. (2024). Home-Based Multi-Sensory and Proximal Strengthening Program to Improve Balance in Charcot-Marie-Tooth Disease Type 1A: A Proof of Concept Study. Muscle Nerve.

[16] Bottoni G., Crisafulli O., Pisegna C., Serra M., Brambilla S., Feletti F., Cremonte G., D’Antona G. (2024). An 8-Month Adapted Motor Activity Program in a Young CMT1A Male Patient. Front. Physiol..

[17] Mori L., Signori A., Prada V., Pareyson D., Piscosquito G., Padua L., Pazzaglia C., Fabrizi G.M., Picelli A., Schenone A. (2020). Treadmill Training in Patients Affected by Charcot-Marie-Tooth Neuropathy: Results of a Multicenter, Prospective, Randomized, Single-Blind, Controlled Study. Eur. J. Neurol..

[18] Tedeschi R., Labanca L., Platano D., Benedetti M.G. (2024). Assessment of Balance During a Single-Limb Stance Task in Healthy Adults: A Cross-Sectional Study. Percept. Mot. Skills.

[19] Jennings M.J., Lochmüller A., Atalaia A., Horvath R. (2021). Targeted Therapies for Hereditary Peripheral Neuropathies: Systematic Review and Steps Towards a ‘Treatabolome’. J. Neuromuscul. Dis..

[20] Luglio A., Maggi E., Riviello F.N., Conforti A., Sorrentino U., Zuccarello D. (2024). Hereditary Neuromuscular Disorders in Reproductive Medicine. Genes.

[21] Pagliano E., Foscan M., Marchi A., Corlatti A., Aprile G., Riva D. (2018). Intensive Strength and Balance Training with the Kinect Console (Xbox 360) in a Patient with CMT1A. Dev. Neurorehabil..

[22] Peters: Joanna Briggs Institute Reviewer’s Manual, JBI—Google Scholar. https://scholar-google-com.ezproxy.unibo.it/scholar_lookup?hl=en&publication_year=2020&author=MDJ+Peters&author=C+Godfrey&author=P+McInerney&author=Z+Munn&author=AC+Tricco&author=H+Khalil&title=Joanna+Briggs+Institute+Reviewer%27s+Manual%2C+JBI.

[23] Tricco A.C., Lillie E., Zarin W., O’Brien K.K., Colquhoun H., Levac D., Moher D., Peters M.D.J., Horsley T., Weeks L. (2018). PRISMA Extension for Scoping Reviews (PRISMA-ScR): Checklist and Explanation. Ann. Intern. Med..

[24] Burns J., Raymond J., Ouvrier R. (2009). Feasibility of Foot and Ankle Strength Training in Childhood Charcot-Marie-Tooth Disease. Neuromuscul. Disord..

[25] Ferraro F., Dusina B., Carantini I., Strambi R., Galante E., Gaiani L. (2017). The Efficacy of Functional Surgery Associated with Early Intensive Rehabilitation Therapy in Charcot-Marie-Tooth Type 1A Disease. Eur. J. Phys. Rehabil. Med..

[26] Mori L., Schenone C., Cotellessa F., Ponzano M., Aiello A., Lagostina M., Massucco S., Marinelli L., Grandis M., Trompetto C. (2022). Quality of Life and Upper Limb Disability in Charcot-Marie-Tooth Disease: A Pilot Study. Front. Neurol..

[27] Tedeschi R. (2023). Kinematic and Plantar Pressure Analysis in Strumpell-Lorrain Disease: A Case Report. Brain Disorders.

[28] Tedeschi R. (2024). Reevaluating the Drucebo Effect: Implications for Physiotherapy Practice. J. Psychosoc. Rehabil. Ment. Health.

[29] Tedeschi R. (2025). The Forgotten DOMS: Recognising Delayed Muscle Soreness in Hand Rehabilitation. Br. J. Sports Med..

